# Supplementary material for: Characterizing the dynamics of the rumen microbiota, its metabolites, and blood metabolites across reproductive stages in Small-tailed Han sheep
Source: Microbiol Spectr. 2023 Nov 10;11(6):e02867-23. doi: 10.1128/spectrum.02867-23 (PMC10715166; doi:10.1128/spectrum.02867-23)
Supplement: Supplemental material — Tables S1 to S3 and Fig. S1 to S7. [file spectrum.02867-23-s0001.docx]

**Table S1** Alpha diversity index at different physiological stages

| Index | Non-pregnancy | Gestation period | Lactation period | *P* value |
| --- | --- | --- | --- | --- |
| ACE | 1195.578±24.69 | 1209.296± 23.12 | 1190.485±14.36 | 0.379 |
| Chao1 | 1202.094±24.20 | 1217.096±20.03 | 1197.415±18.81 | 0.340 |
| Simpson | 0.989±0.00 | 0.988±0.00 | 0.988±0.01 | 0.993 |
| Shannon | 8.183±0.26 | 8.045±0.33 | 8.165±0.37 | 0.769 |

Note: Peer data shoulders marked with different lowercase letters indicate significant differences (*P* < 0.05), the same letters or no letters indicate non-significant differences (*P* > 0.05).

**Table S2** Comparison of differences in rumen flora phylum levels at different physiological stages (%)

| Parameter | Non-pregnancy | Gestation period | Lactation period | *P* value |
| --- | --- | --- | --- | --- |
| Chloroflexi | - | 0.01 | 0.02 | ＜0.001 |
| Fibrobacteres | - | 0.04 | 0.16 | 0.008 |
| Lentisphaerae | - | 0.01 | 0.01 | 0.029 |
| Spirochaetes | - | 0.47 | 1.03 | 0.040 |
| Bacteroidetes | - | 50.30 | 45.70 | 0.113 |
| Atribacteria | 0.02 | - | 0.04 | 0.007 |
| Fibrobacteres | 0.07 | - | 0.16 | 0.040 |

**Table S3** Comparison of differences in rumen flora genus levels at different reproductive stages (%)

| Parameter | Non-pregnancy | Gestation period | Lactation period | *P* value |
| --- | --- | --- | --- | --- |
| *Prevotella_1* | - | 31.50 | 19.80 | 0.002 |
| *Moryella* | - | 0.26 | 0.42 | 0.008 |
| *Lachnoclostridium* | - | 0.00 | 0.02 | 0.009 |
| *Fibrobacter* | - | 0.04 | 0.16 | 0.010 |
| *Treponema_2* | - | 0.40 | 0.90 | 0.041 |
| *Ruminococcus_2* | 2.49 | 5.94 | - | 0.043 |
| *uncultured_bacterium_f_Lachnospiraceae* | 3.37 | 4.44 | - | 0.036 |
| *[Eubacterium]_ruminantium_group* | 0.77 | - | 1.21 | 0.005 |
| *Moryella* | 0.27 | - | 0.42 | 0.037 |
| *Fibrobacter* | 0.07 | - | 0.16 | 0.043 |
| *Helicobacter* | 0.06 | - | 0.03 | ＜0.001 |
| *Erysipelotrichaceae_UCG-009* | 0.15 | - | 0.05 | 0.015 |


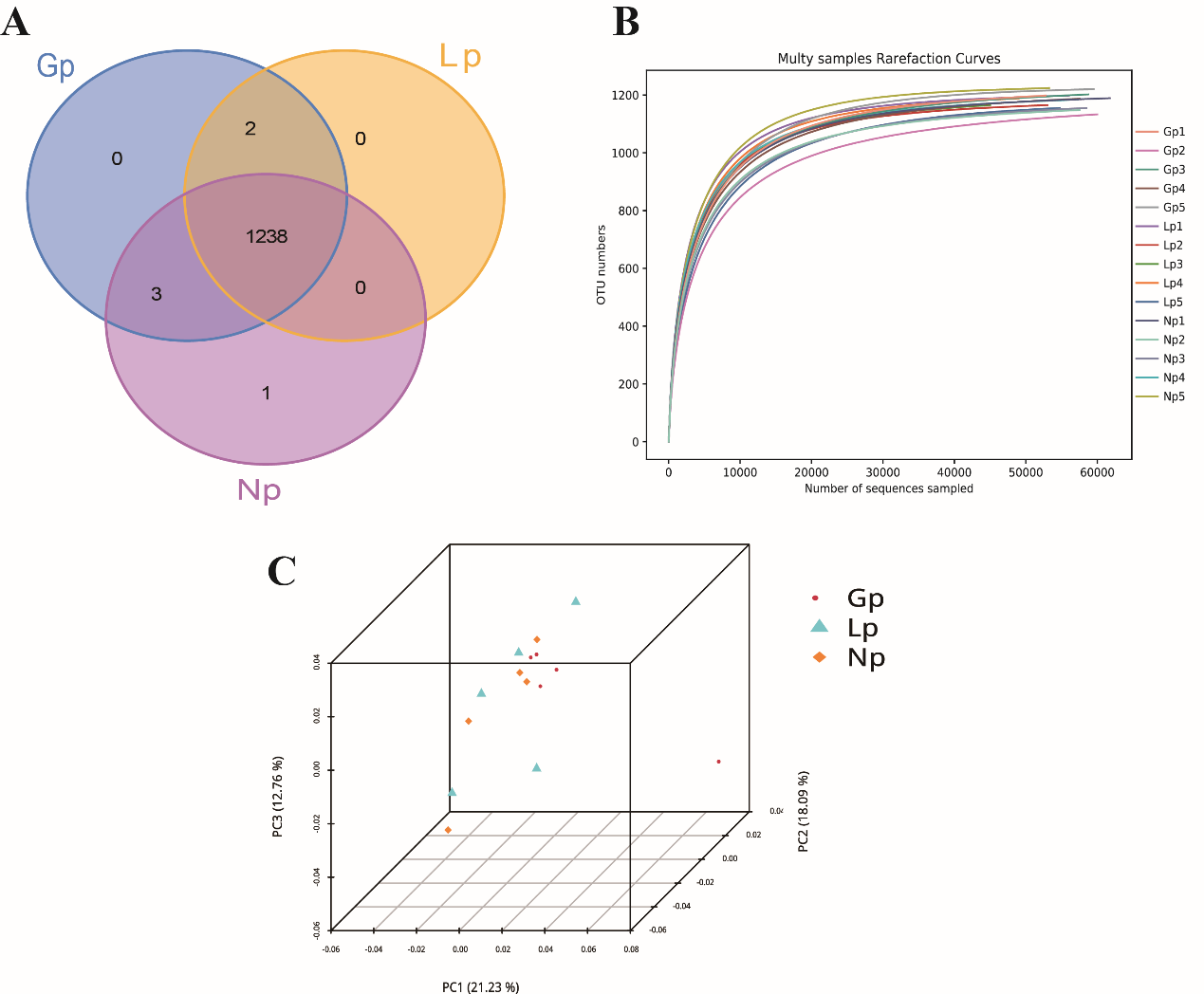


**Figure S1.** Analysis of the rumen microbiota diversity. (A) Distribution of OTU; (B) dilution curve; (C) PCoA. NP: Non-pregnancy, Gp: Pregnancy, LP: Lactation.

**Figure S2.** Up-down-regulated differential microbiota metabolites in the top 10 differential multiples


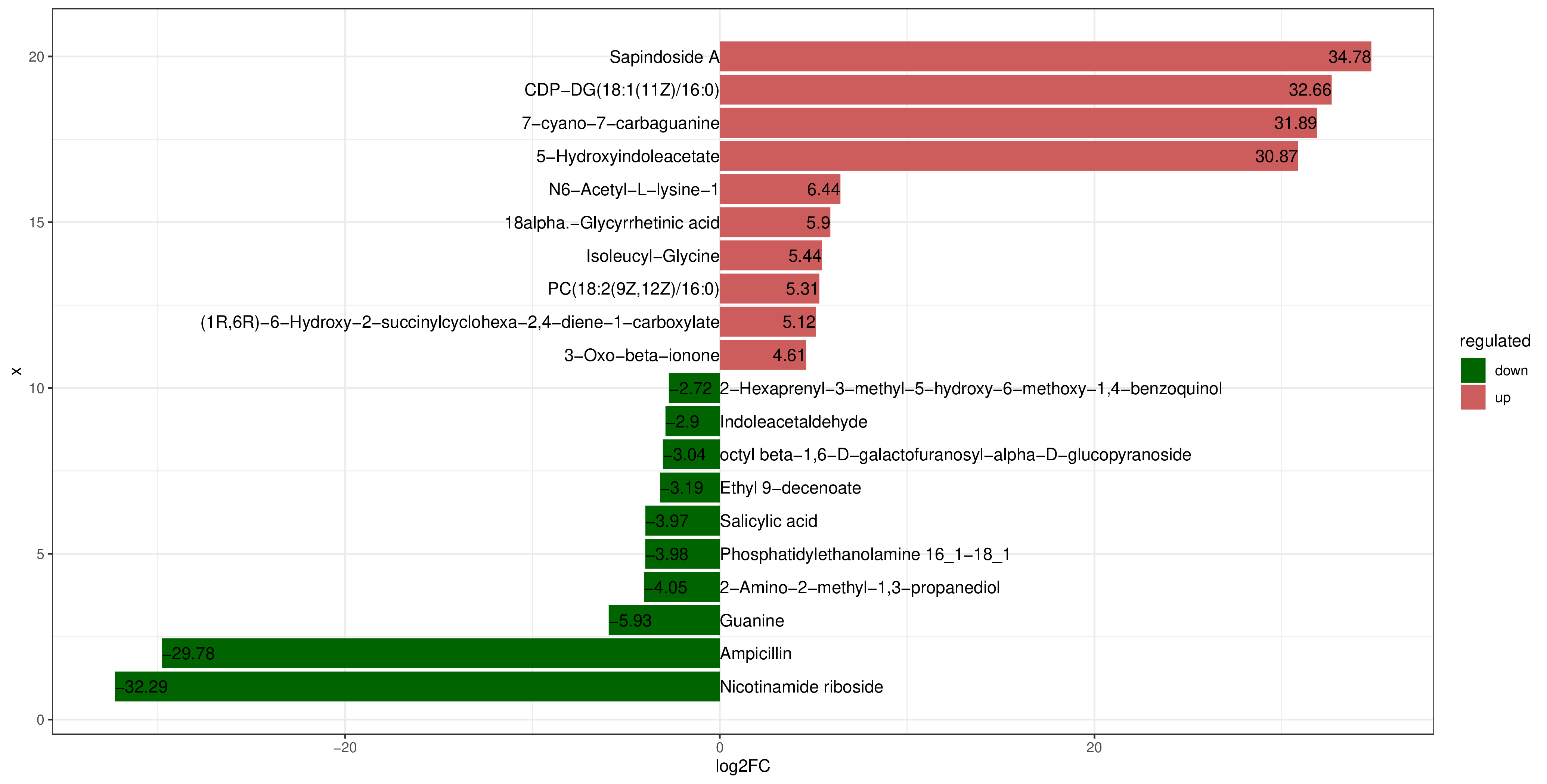


NP-GP


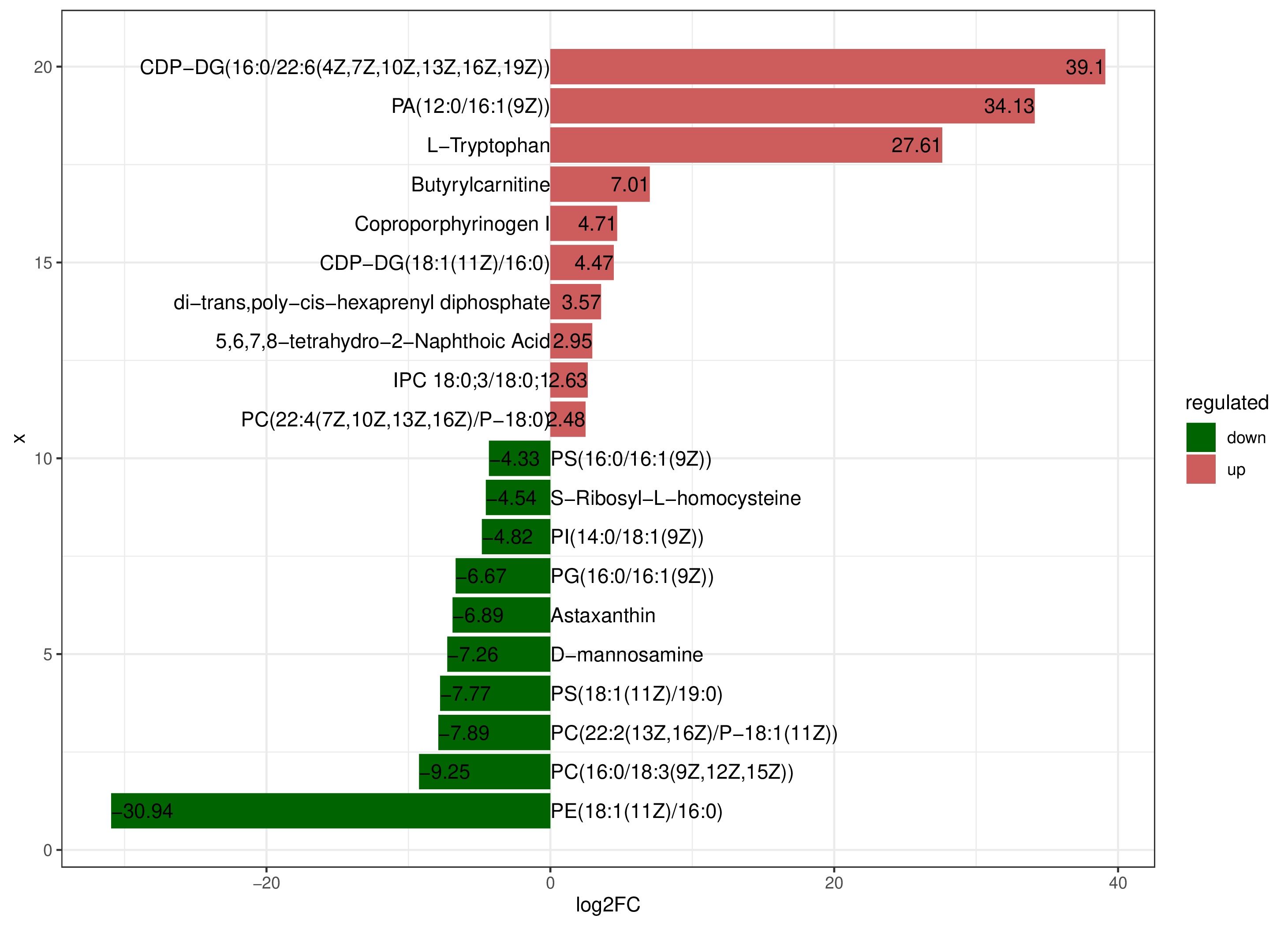


GP-LP


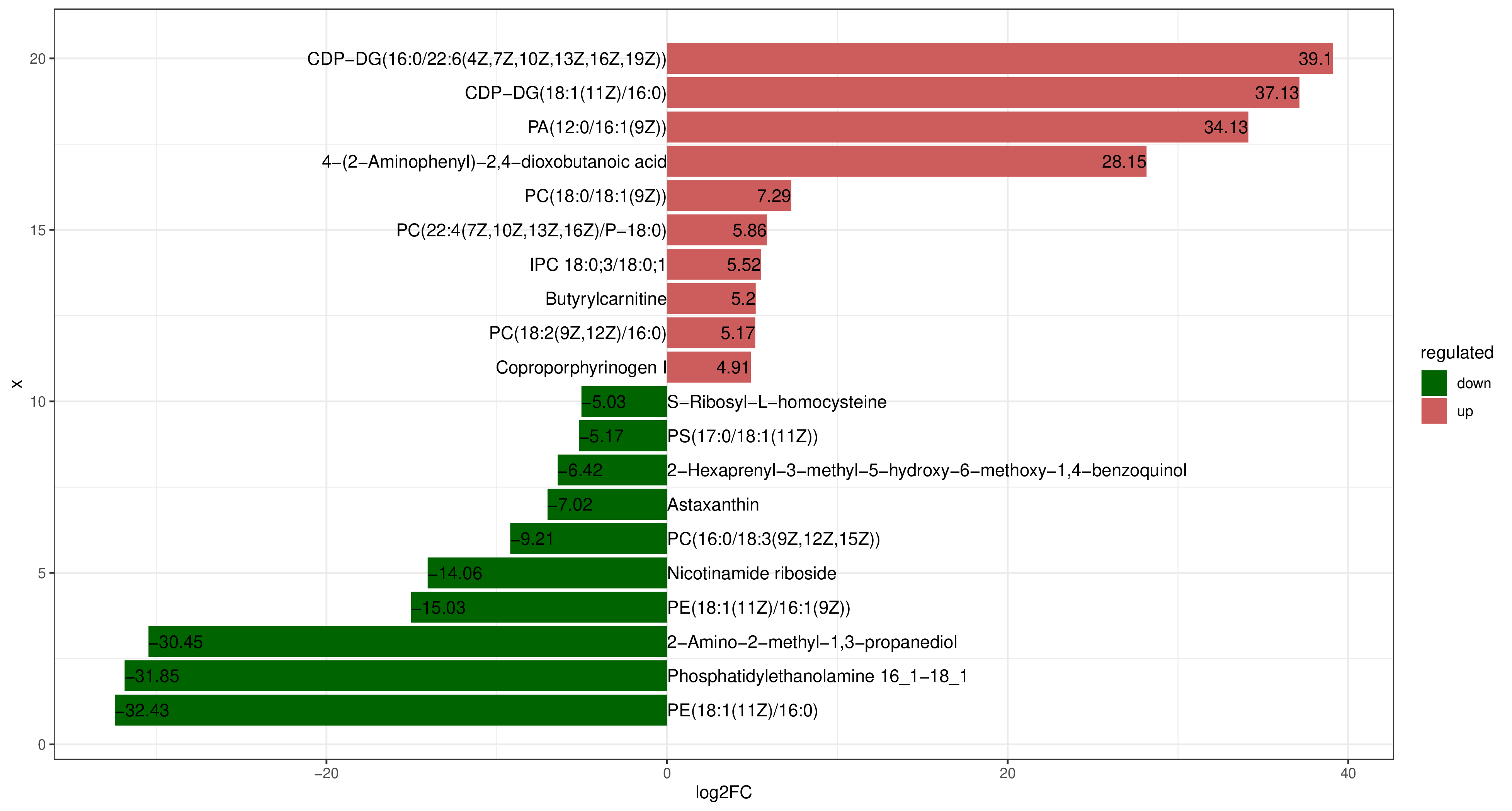


NP-LP

**Figure S3.** Cluster analysis of microbial metabolites


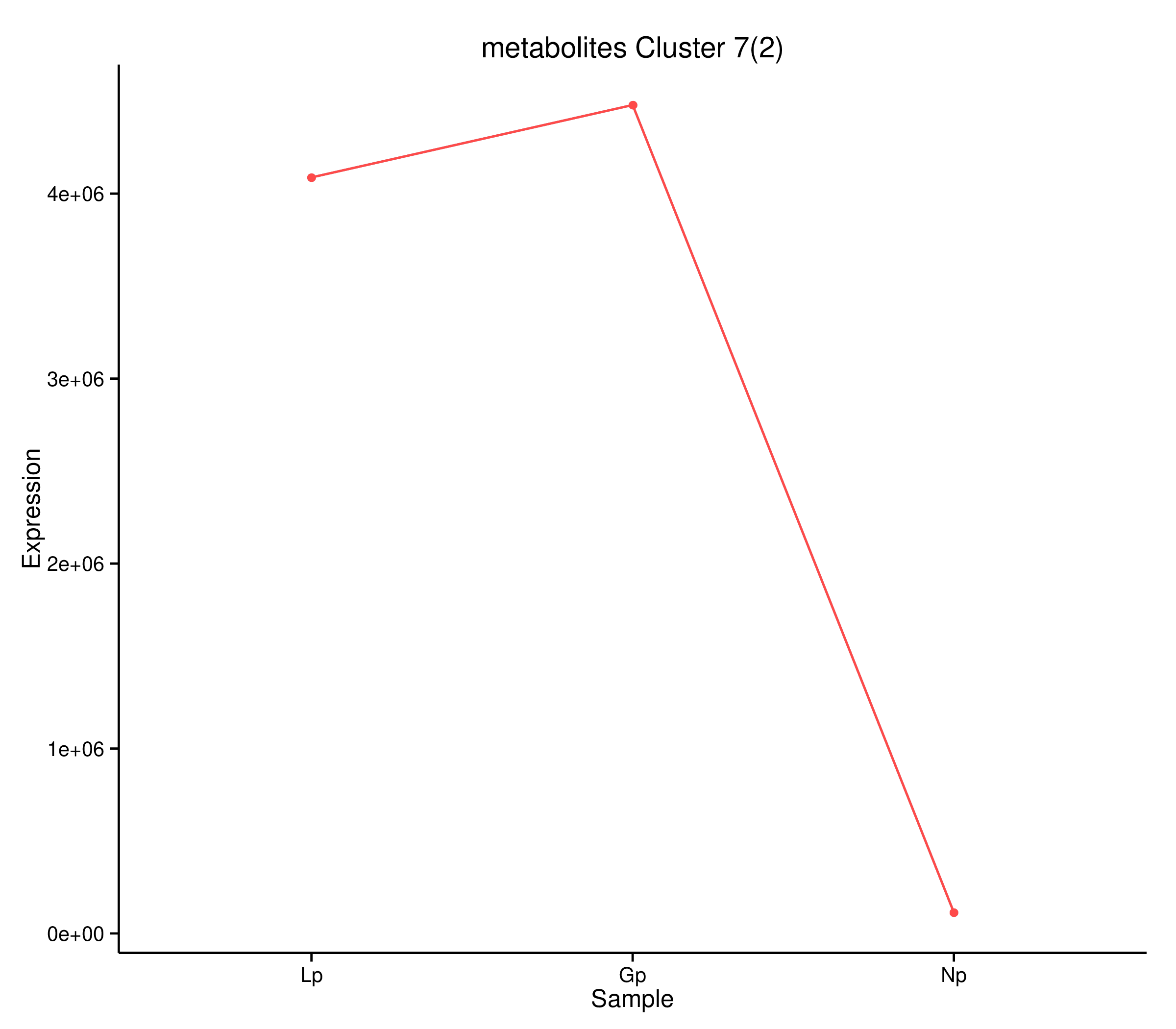
 PC(18:2(9Z,12Z)/16:0)


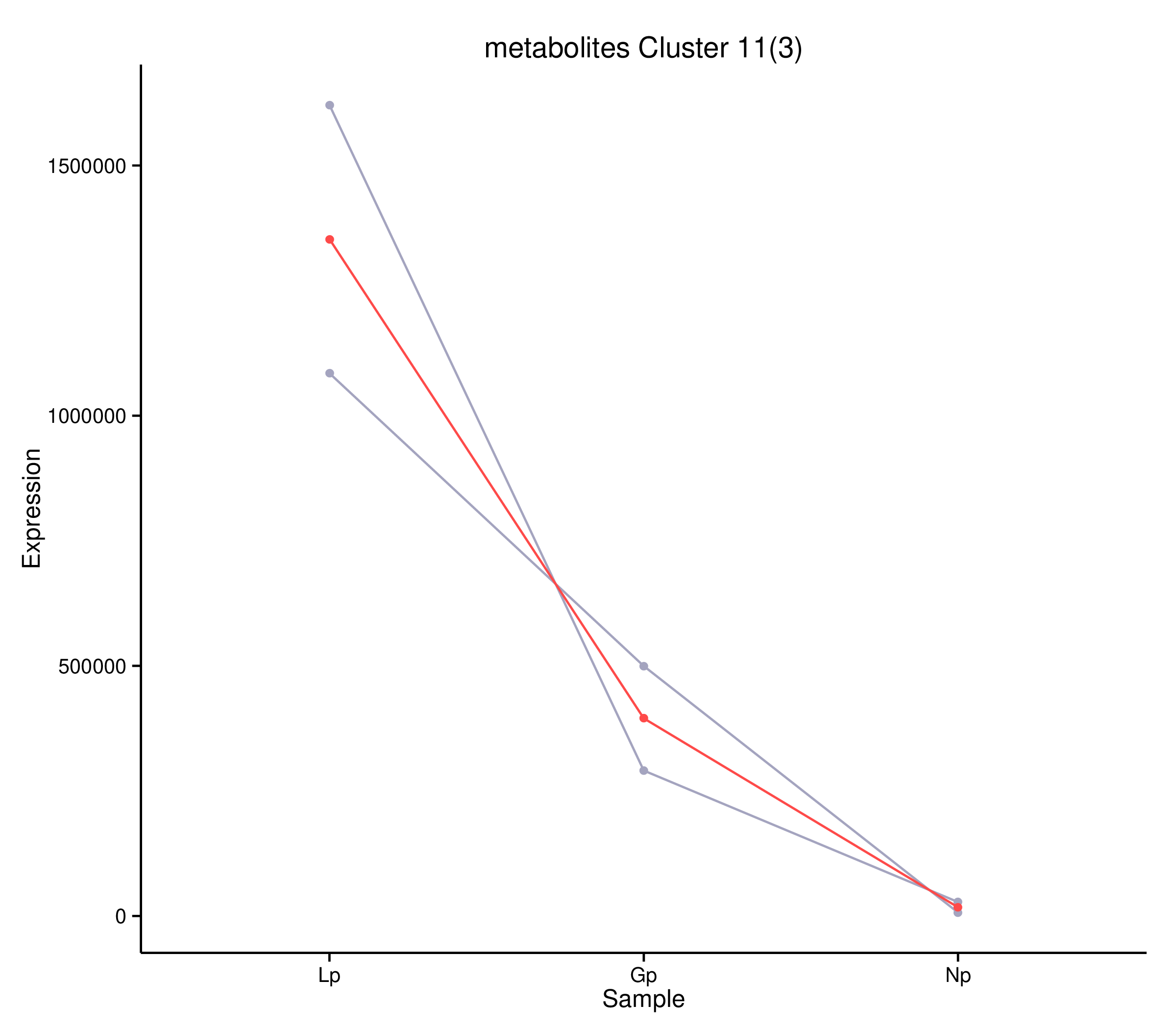


PC(18:0/18:1(9Z))、PC(22:4(7Z,10Z,13Z,16Z)/P-18:0)


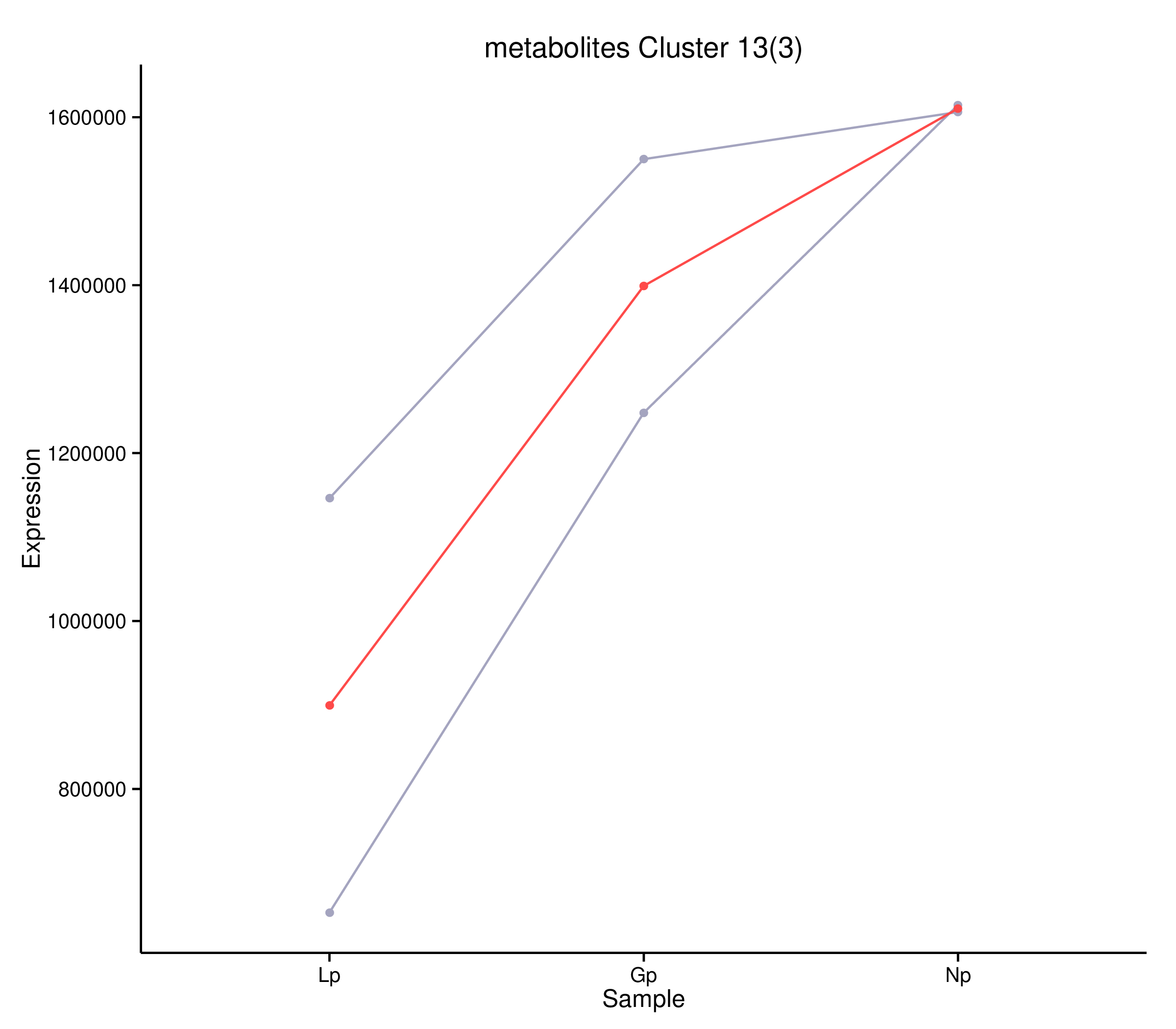


1-Stearoyl-2-hydroxy-sn-glycero-3-phosphocholine、β-Cryptoxanthin


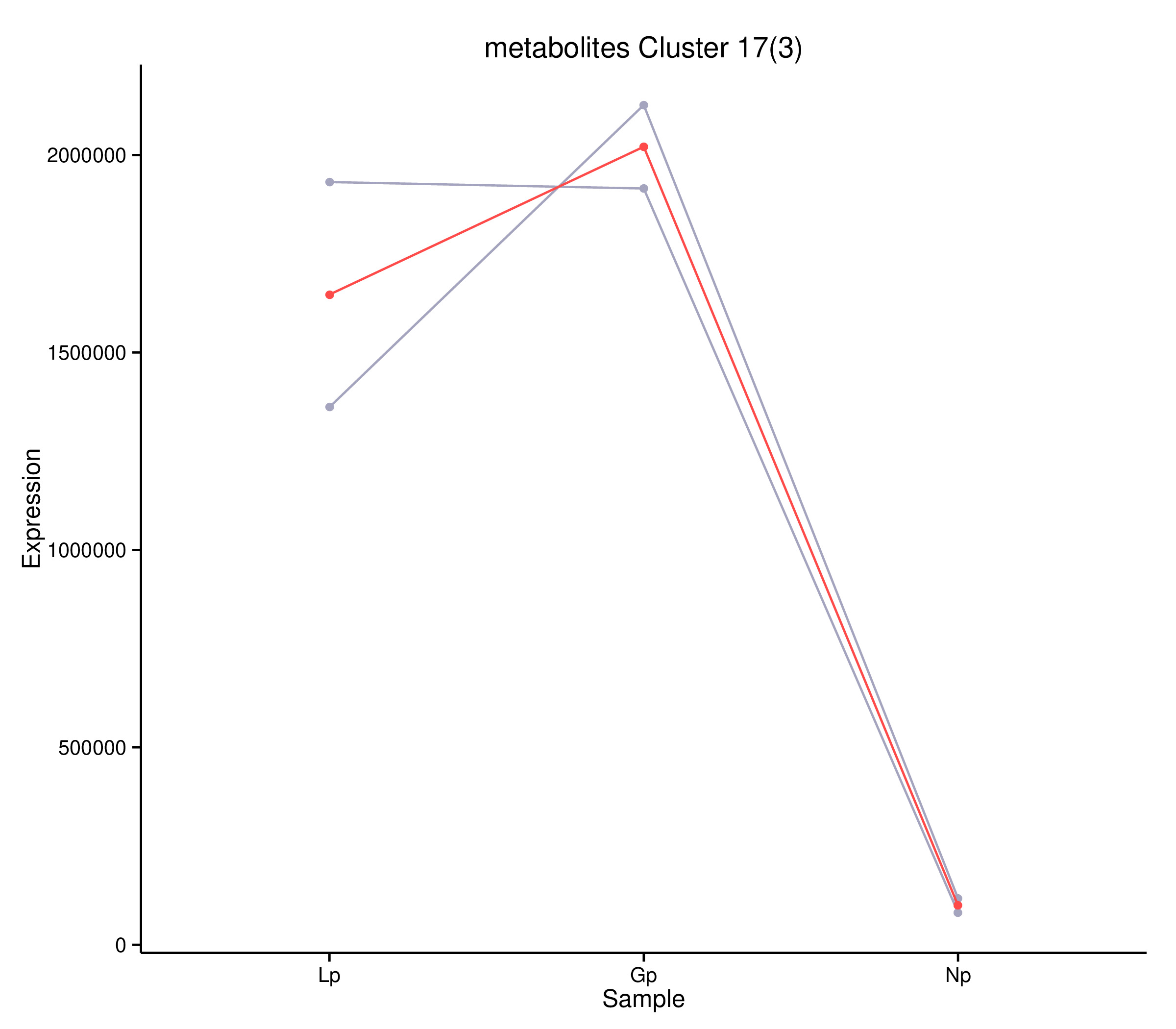


PE(18:1(11Z)/19:0)、PC(16:0/18:0)


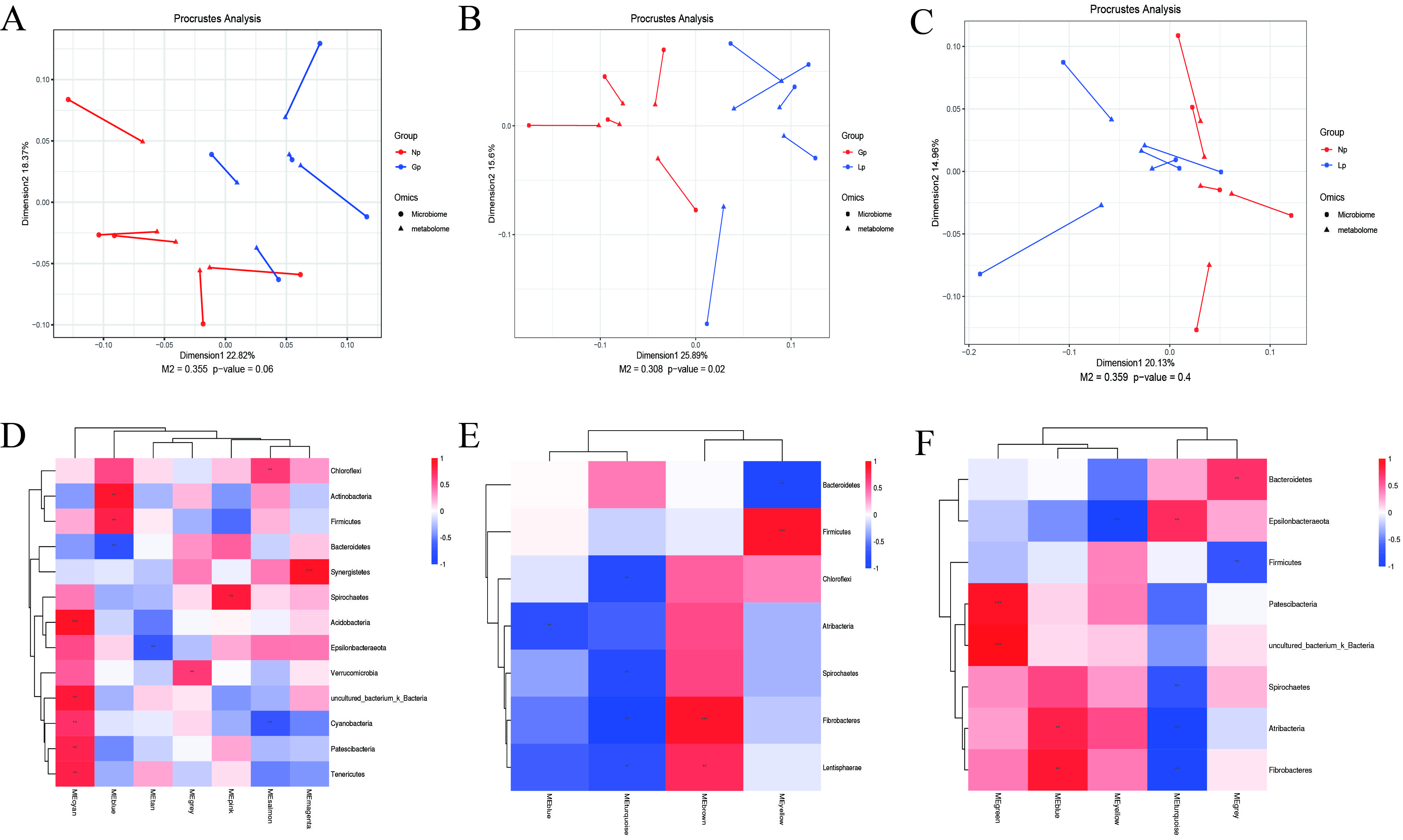


**Figure S4.** Combined analysis of the rumen microbiome and metabolites. A-C: Microbial-metabolite Procrustes analysis; D-F: Microbial-metabolite correlation heatmap. Note: A, D: NP-GP; B, E: GP-LP; C, F: NP-LP. *, ** and *** indicate *P* < 0.05, *P* < 0.01 and *P* < 0.001, respectively. NP: Non-pregnancy, Gp: Pregnancy, LP: Lactation.

**Figure S5.** Heat map of correlation between differential metabolites and genus level microbiota


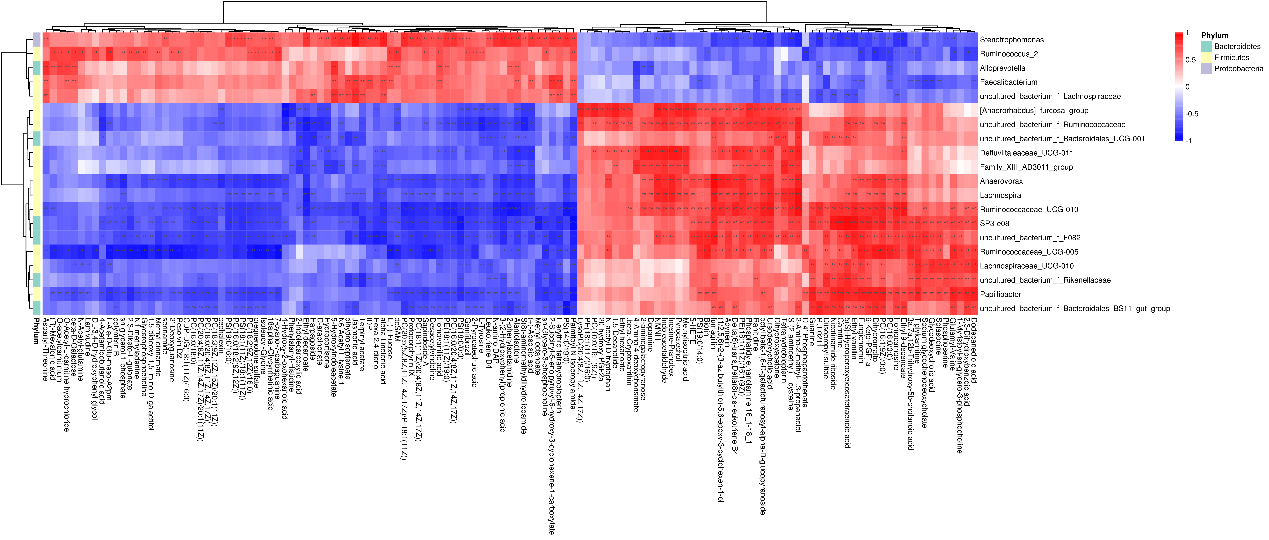


NP-GP


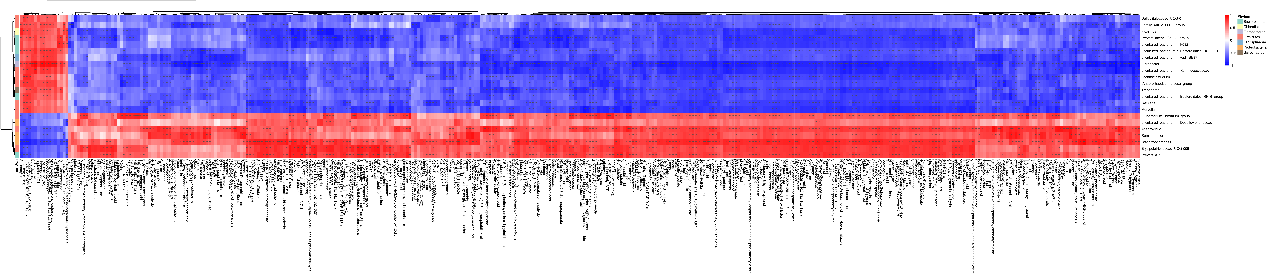


GP-LP


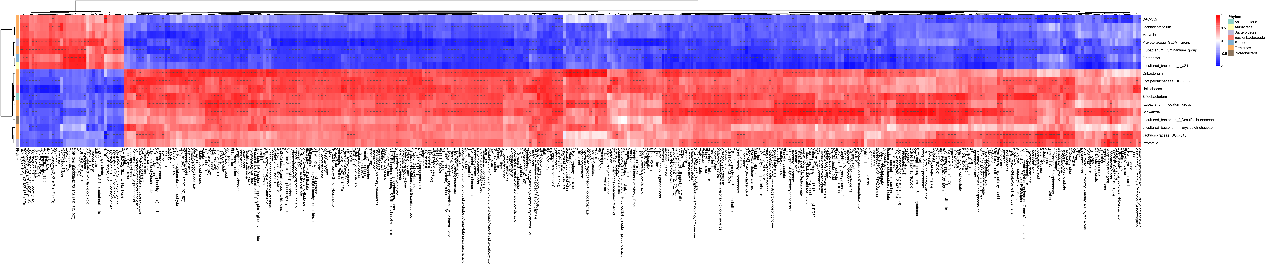


NP-LP

**Figure S6.** up-down-regulated differential blood metabolites in the top 10 differential multiples


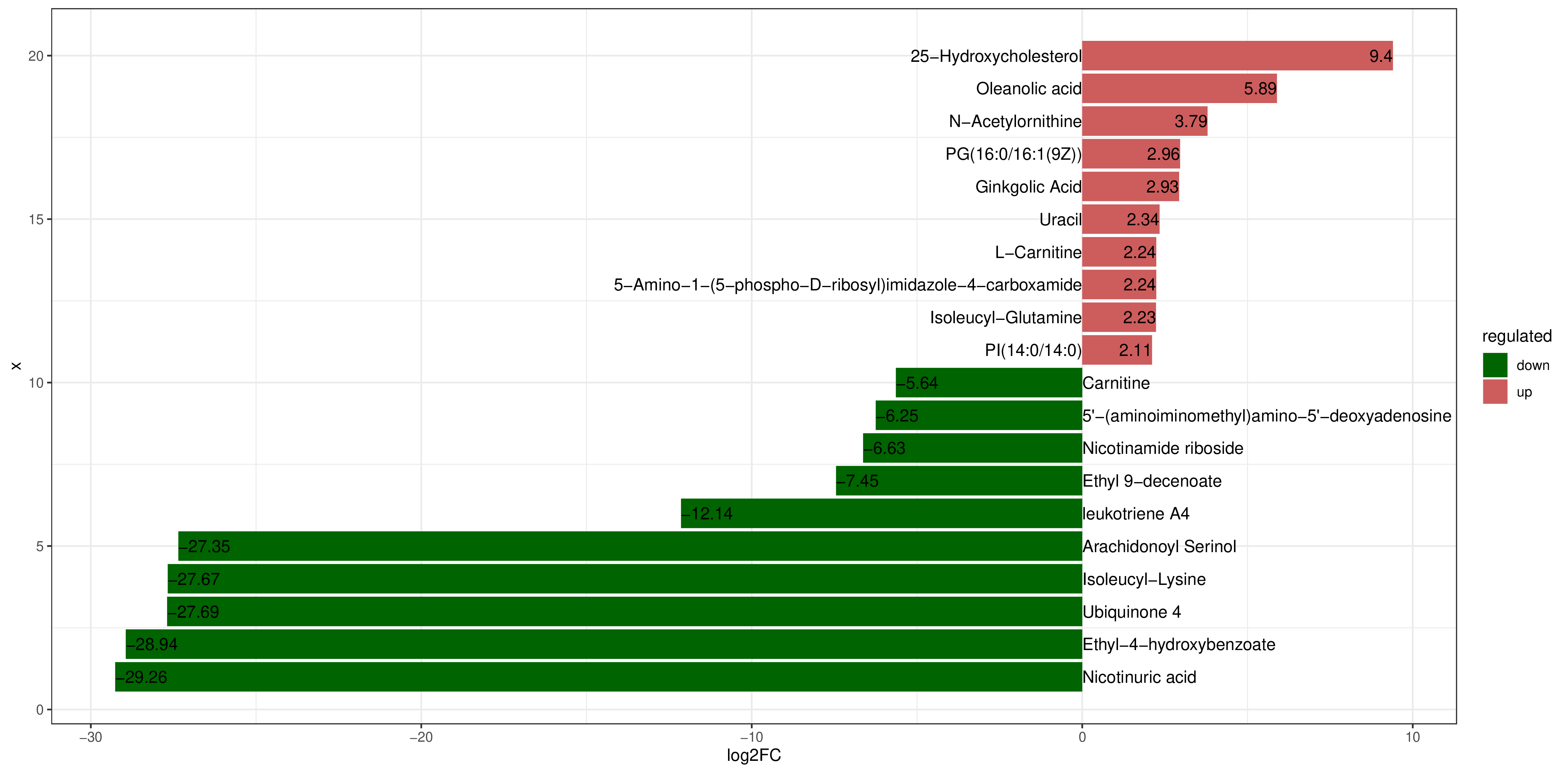


NP-GP


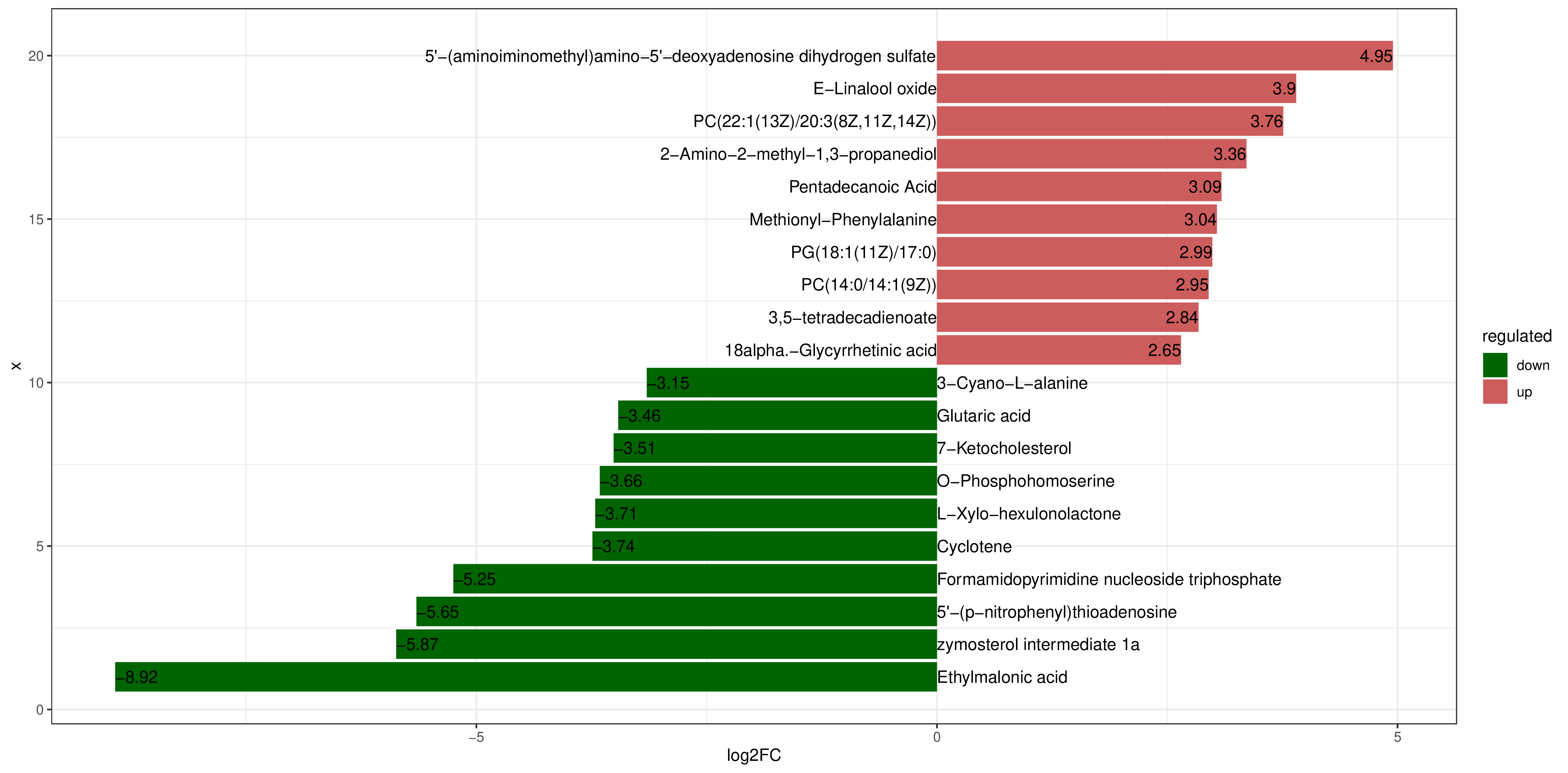


GP-LP


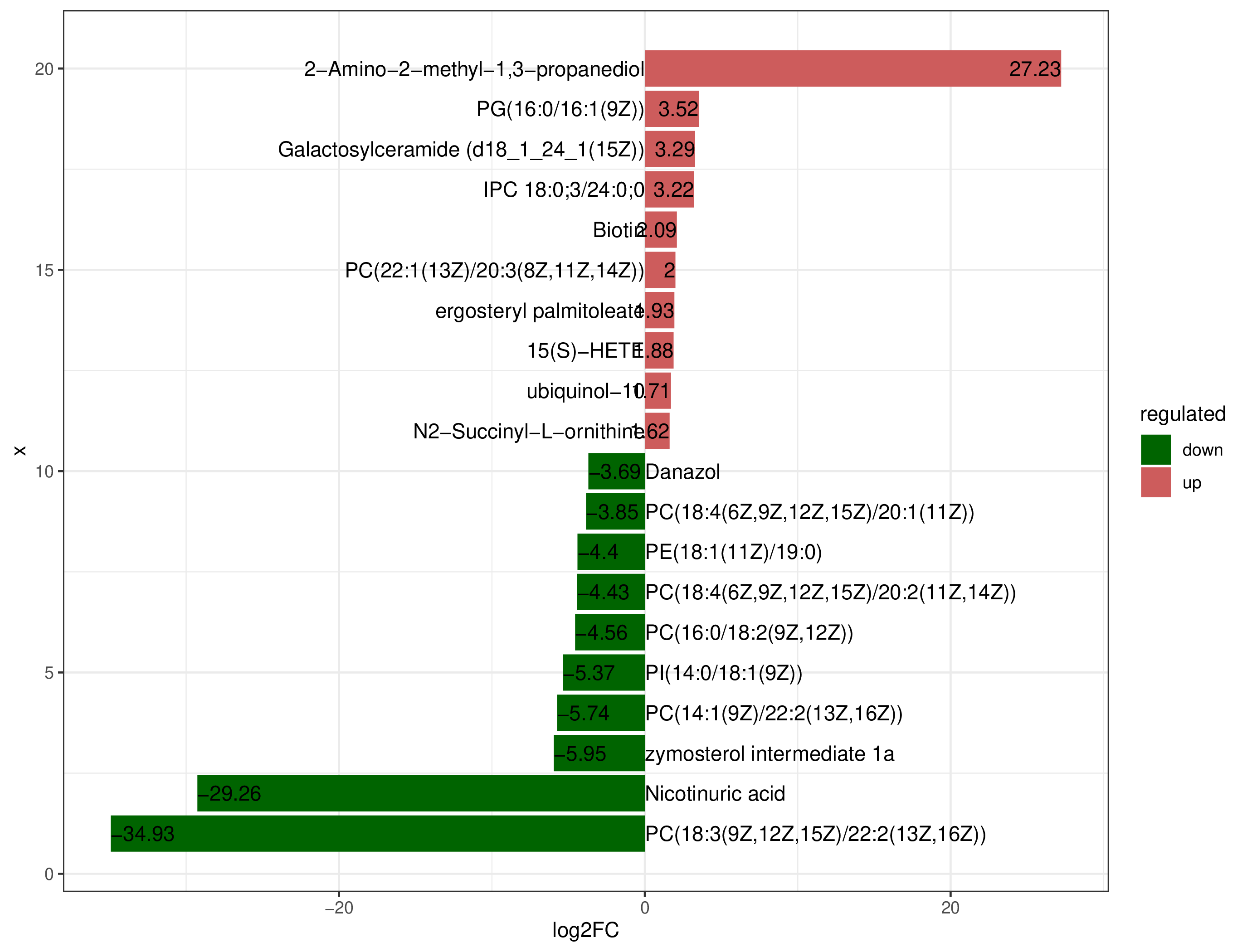


NP-LP

**Figure S7.** Cluster of metabolites of blood differences


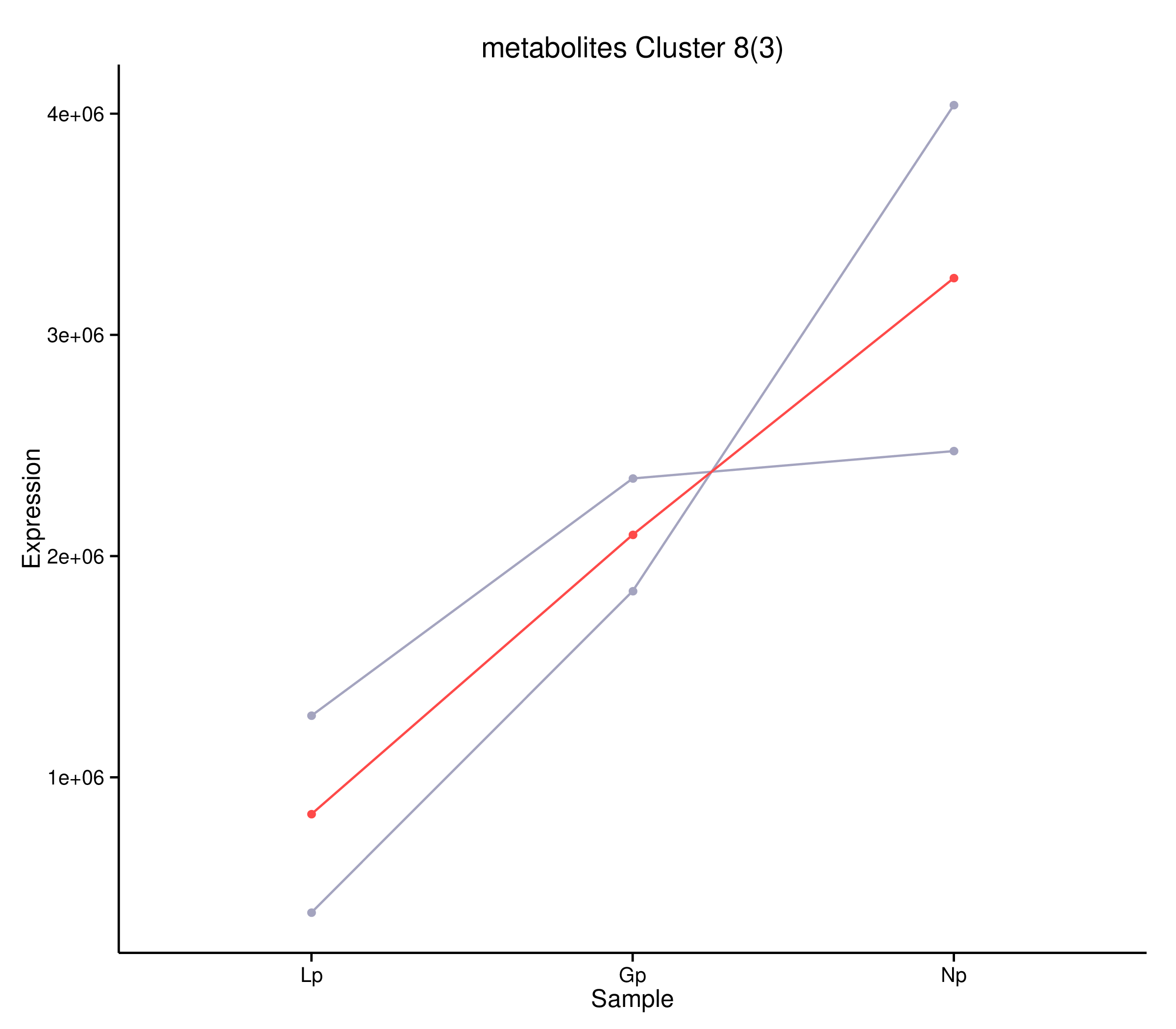


PC(18:2(9Z,12Z)/16:0)、 PC(22:4(7Z,10Z,13Z,16Z)/P-18:0)


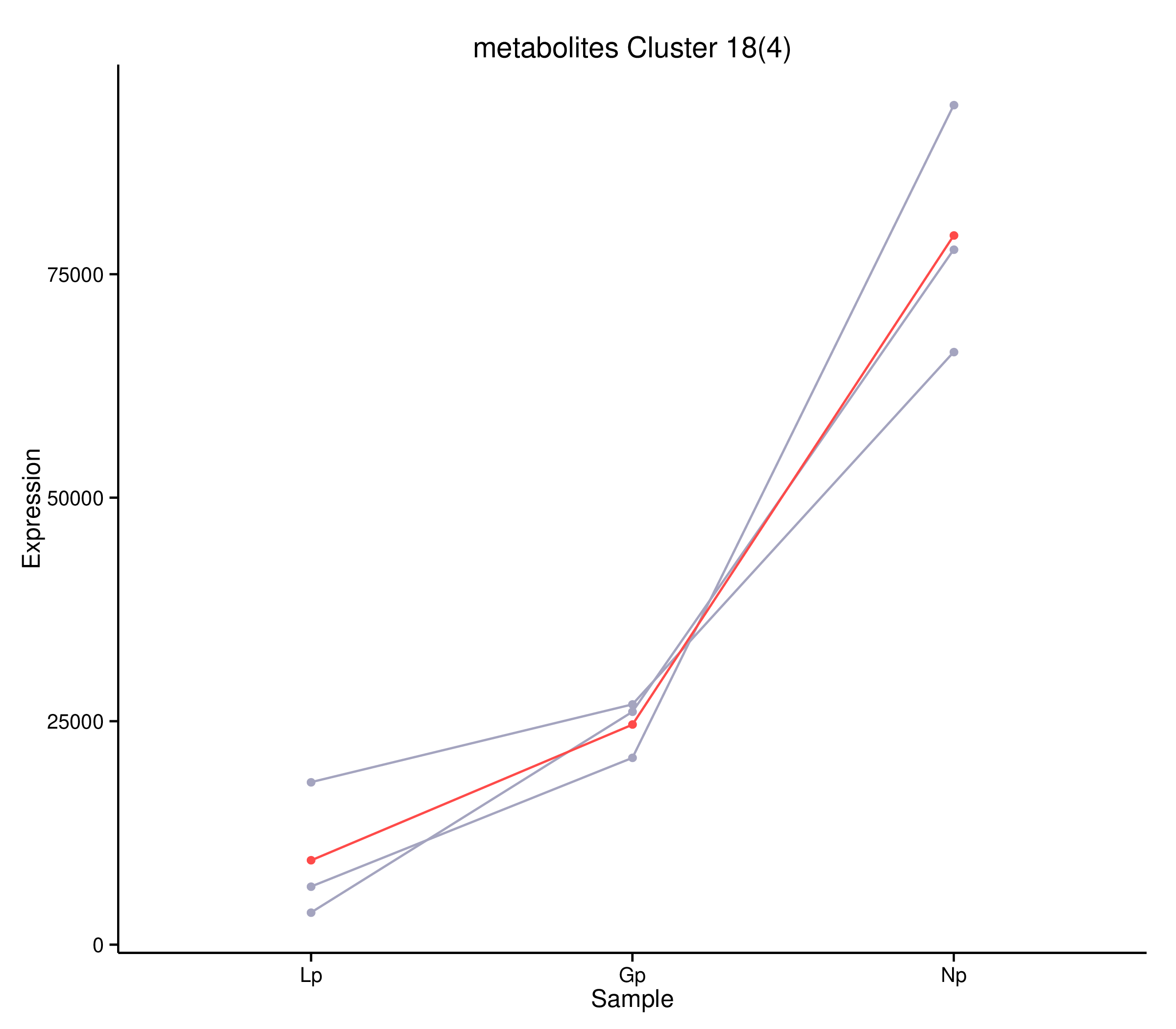


PC(18:4(6Z,9Z,12Z,15Z)/20:1(11Z))、PC(18:4(6Z,9Z,12Z,15Z)、20:2(11Z,14Z))


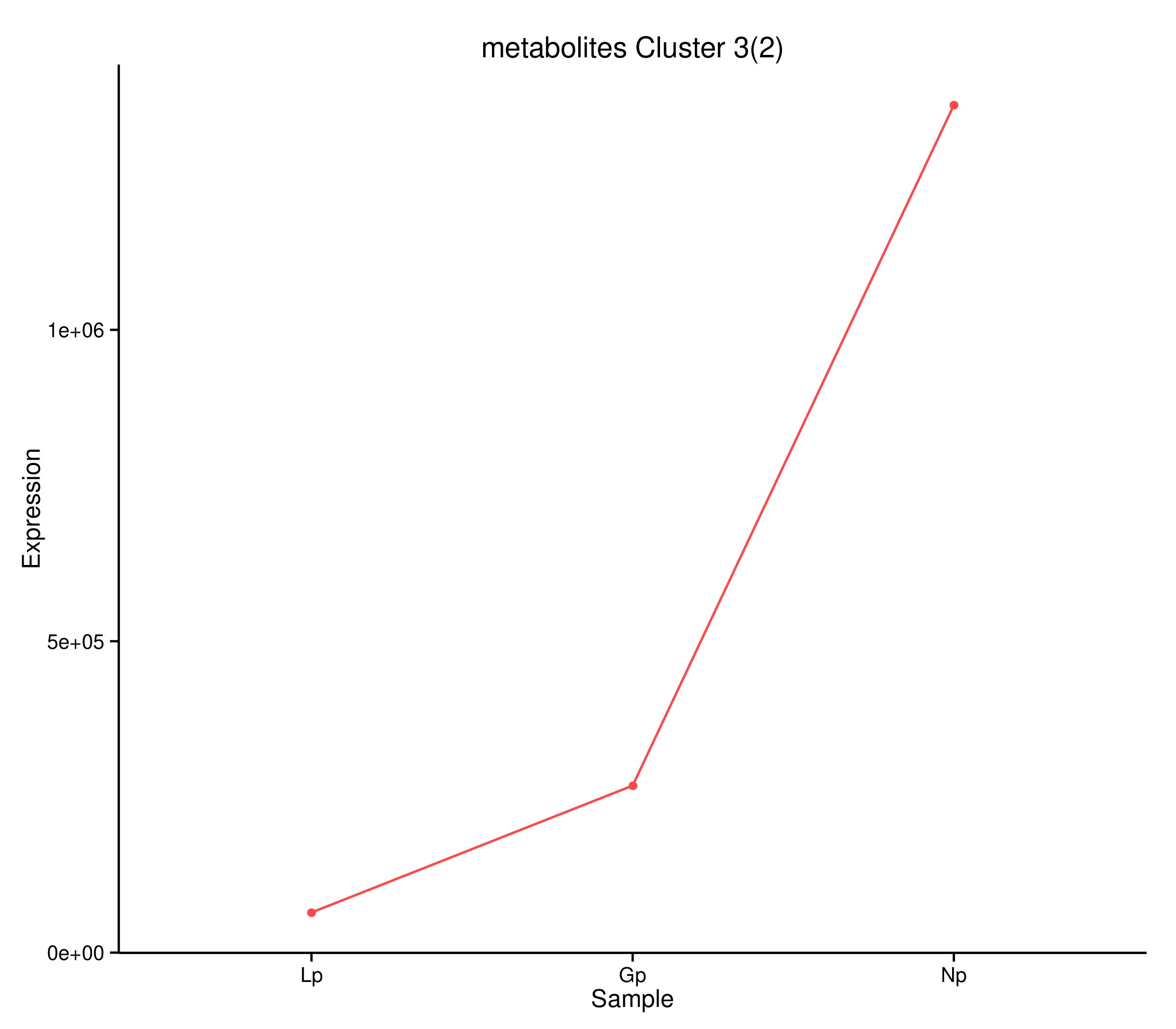
 PE (18:1(11Z)/19:0)
